# *Paraclostridium bifermentans* exacerbates pathosis in a mouse model of ulcerative colitis

**DOI:** 10.1371/journal.pone.0197668

**Published:** 2018-05-21

**Authors:** Ryo Kutsuna, Junko Tomida, Yuji Morita, Yoshiaki Kawamura

**Affiliations:** Department of Microbiology, Aichi Gakuin University Graduate School of Pharmaceutical Science, Nagoya, Aichi, Japan; Cincinnati Children's Hospital Medical Center, UNITED STATES

## Abstract

Although it has been recognized that intestinal bacteria play an important role in the pathology of human ulcerative colitis (UC), specific pathogenic bacteria for UC have not been identified. We investigated the influence of *Paraclostridium bifermentans* PAGU1678 strain on the pathology of a UC mouse model and found it increased UC pathosis scores such as loose and bloody stools, reduced diversity of fecal flora, disappearance of the crypt structure of distal colon tissue, destruction of intestinal epithelial cells, and atrophy of the colon. Furthermore, we observed an increase in *COX-2*, *TNF-α*, *IL-6*, *IL-1*, and *IL-17* expression and a decrease in *Foxp3* and *SOCS3* expression, as inflammation-related factors and inflammatory cytokines, a decrease in the concentration of short chain fatty acids (acetic acid, propionic acid, and butyric acid) in feces, and an increase of intestinal mucosal myeloperoxidase activity. These results suggest that *P*. *bifermentans* PAGU1678 is a pathology-exacerbating factor in a mouse model of UC. This study is the first to demonstrate exacerbation of the pathological condition in a mouse model of UC by a single bacterial strain.

## Introduction

Ulcerative colitis (UC) and Crohn’s disease, which are classified as inflammatory bowel disease (IBD), are characterized by symptoms such as chronic and recurrent bloody diarrhea and abdominal pain [1, 2]. Although it is suggested that environmental factors, such as food and stress in daily life, and genetic factors are causative agents of UC, the underlying mechanisms are not understood completely [3–5]. However, it has been reported that some intestinal bacteria have an influence on UC pathology. A group of sulfate-reducing bacteria was increased by approximately 2-fold in the colon of UC patients in the active phase compared to healthy subjects and UC patients in the remission phase [6–9]. In addition, *Fusobacterium varium* was demonstrated to attach to and invade the mucosa of the colon, promoting the production of inflammatory cytokines, such as IL-8 and TNF-α, from the mucosa [10].

As for the intestinal bacterial flora, in the colon of IBD patients, dysbiosis associated with a reduction of the phylum *Firmicutes* including the class *Clostridia*, an increase of the genus *Bacteroidetes* and family *Enterobacteriaceae*, and a reduction of the total number of bacteria has been reported [11–13]. Furthermore, since colonization of the mouse colon with a mixture of *Clostridium* species derived from the colon or a single strain of *Clostridium butyricum* showed an inhibitory effect on mouse colitis via the induction of IL-10, the class *Clostridia* is generally considered to help maintain health [14–16].

Our metagenomic analysis revealed that a certain *Clostridium* sp. was significantly increased in feces from a mouse model of UC compared with a healthy control group [17]. The 16S rRNA gene of the identified *Clostridium* sp. showed the highest similarity (>99%) to the 16S rRNA gene of *Paraclostridium bifermentans*, and was re-classified from the genus *Clostridium* to the genus *Paraclostridium* [18]. *P*. *bifermentans* has also been found in human intestinal flora and shown to be involved in some diseases, such as metastatic osteomyelitis, necrotizing pneumonia, and bacteremia; however, there have been no reports of its association with UC [19–21]. In the present study, we examined the effects of *P*. *bifermentans* PAGU1678 on the pathology of a mouse model of UC.

## Materials and methods

### Bacterial strains, experimental animal models, and bacterial suspension treatment

*P*. *bifermentans* PAGU1678 (received as *Clostridium* sp. ID4, isolated from a rat fecal sample, directly from Dr. M. Kalmokoff) [22] was used as well as two control strains (*C*. *butyricum* PAGU1417^T^ = GTC1351^T^ and *Lactobacillus plantarum* PAGU1415^T^ = GTC1709^T^), both of which are known to improve UC pathosis [14, 23, 24]. These strains were incubated at 37°C for 24 h under anaerobic conditions (10% CO_2_, 10% H_2_, 80% N_2_) on GAM agar medium (NISSUI, Tokyo, Japan). The cells were suspended in sterilized phosphate-buffered saline (PBS) to OD_600_ = 1.0 (corresponding to 2.0 × 10^9^ colony-forming units) for oral administration.

**Fig 1:**
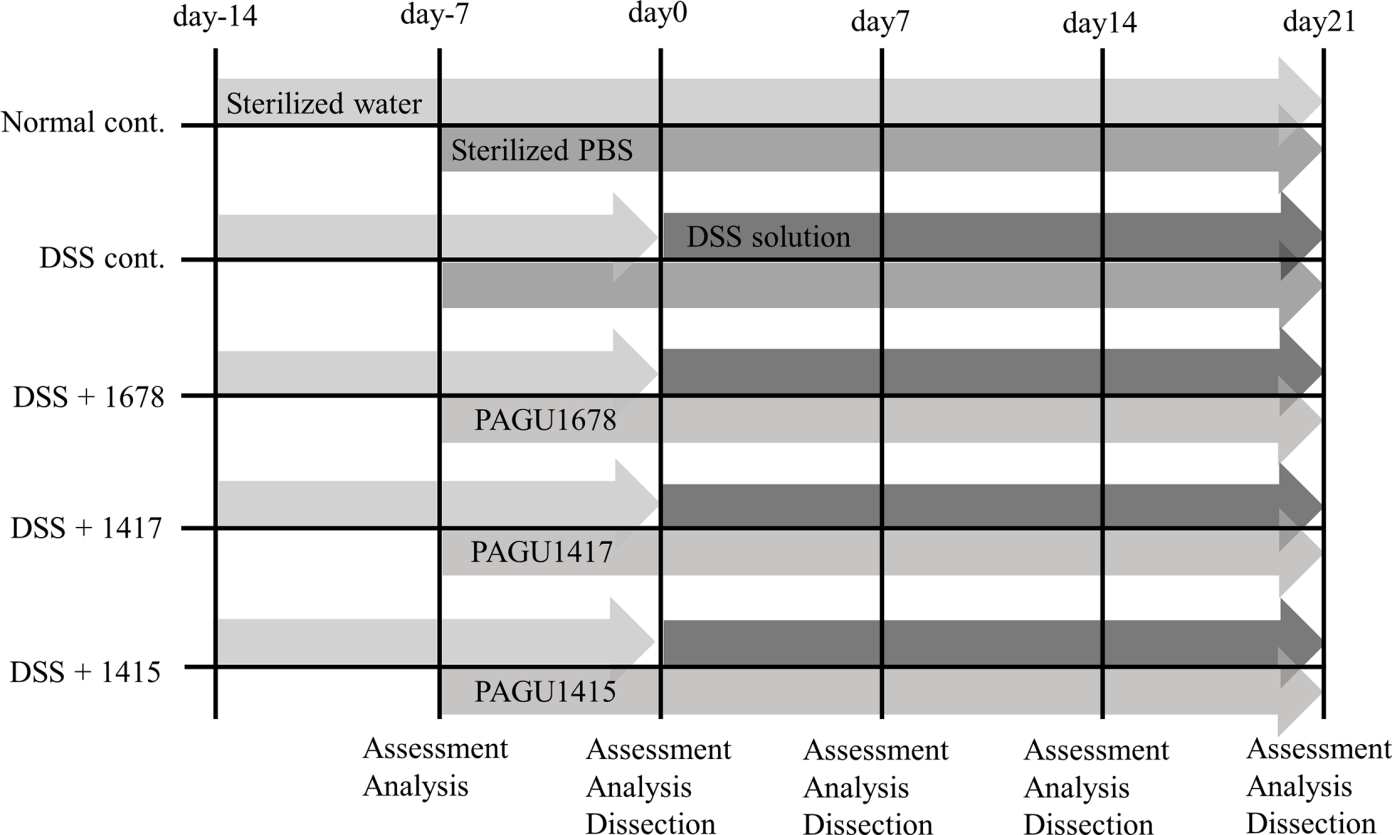
Experimental design. The start date of DSS administration was defined as day 0. C57BL/6J mice were randomized into 5 groups (*n* = 12/group), and each group was given sterile water and adaptation for 1 week. From day -7, each bacterial suspension or sterilized PBS treatment was started while free drinking of sterilized water was continued. From day 0, sterilized water was switched to DSS (1%) and colitis was induced. Weight measurement and fecal sample collection were carried out every week from day -7 to day 21, and further from day 0 to day 21; the assessment of inflammation and dissection of 3 animals per group were also performed.

Five-week-old female inbred C57BL/6J mice (Japan SLC, Shizuoka, Japan) were housed in a room maintained at a standard condition (22°C, 12 h light/dark cycle) throughout the experiment. The mice were allowed free access to a standard mouse chow diet (MF; Oriental Yeast, Tokyo, Japan) and sterile drinking water. After adaptation for 1 week, the mice were randomized into 5 groups (*n* = 12/group): healthy group (Normal cont.), dextran sulfate sodium (DSS) (1% w/v: molecular weight 5000; Wako Pure Chemical Industries, Osaka, Japan) -treated group (DSS cont.), DSS- (1%) and PAGU1678-treated group (DSS+1678), DSS- (1%) and PAGU1417-treated group (DSS+1417), and DSS- (1%) and PAGU1415-treated group (DSS+1415) (Fig 1). Oral administration (0.2 mL/mouse/day) of each bacterial suspension was started at 1 week before DSS treatment (day -7). For the Normal cont. and DSS cont. groups, sterilized PBS (0.2 mL/mouse/day) was administered instead of the bacterial suspension. After 1week, experimental colitis was induced by the oral administration of DSS (1%; day 0). During treatment with DSS (1%), we continued the oral administration of each bacterial suspension or PBS once per day. All animal experiments were performed in accordance with the Regulations on Animal Experimentation at the School of Pharmacy, Aichi Gakuin University (Nagoya, Aichi, Japan). All procedures to maintain and use the mice were approved by the Animal Care and Use Committee of the School of Pharmacy, Aichi Gakuin University (Nagoya, Aichi, Japan) (permission number: 17–018). All surgery was performed under diethyl ether anesthesia, and all efforts were made to minimize suffering.

### Assessment of inflammation in DSS-induced colitis and sample collection for each analysis

In order to evaluate the severity of DSS-induced colitis, the weight and fecal condition of each mouse were recorded every 7 days based on the disease activity index (DAI; Table 1). DAI score was based on the index of M S Islam et al., and modified it for observation in more detail [25]. Then, each mouse was anesthetized and its entire colon was removed from the cecum to the anus. Its length was measured using a ruler and then washed with sterilized PBS to remove any remaining stool content. A part of the distal colon was frozen in liquid nitrogen, and stored at -80°C until use. The remaining colon (2/3 of the whole colon) was embedded in Tissue-Tek O.C.T. Compound (Sakura Finetek, Torrance, CA, USA), frozen in liquid nitrogen, and stored at -80°C until use.

**Table 1:** DAI.

Score	Stool softness	Bloody stool	Body weight loss	Activity	Fur condition
0	Normal	Normal	0%	Active	Normal
1	Soft	Part of the stool	5%	Walking	Mildly fluffy
2	Mild diarrhea	—	—	—	—
3	Severe diarrhea	All of the stool	10%	Motionless	Severely fluffy

The DAI score of mouse colitis was evaluated using stool condition (softness, bloody), body weight loss from peak, and appearance (activity, fur condition). Scoring of each mouse was carried out using a scale of 0 to 3 points according to severity.

### Stool sample collection and DNA extraction

Fecal samples were collected from each mouse and stored at -30°C until use. Feces were suspended in sterilized water using a grinder pestle (SANSYO, Tokyo, Japan). After residual mass was removed, genomic DNA was extracted using a MORA EXTRACT Kit (AMR, Gifu, Japan). Extracted DNA was stored at -30°C until use.

### PCR amplification and denaturing gradient gel electrophoresis (DGGE) analysis

16S rRNA genes from mouse fecal DNA were amplified using a bacterial universal primer pair set: 341F (GC-clamp) primer (5′-CGC CCG GGG CGC GCC CCG GGC GGG GCG GGG GCA CGG GGG GAC TCC TAC GGG AGG CAG CAG T-3′) and 518R primer (5′-ATT ACC GCG GCT GCT GG-3′) [26]. PCR was performed with a 2720 Thermal Cycler (Applied Biosystems, Foster City, CA, USA) using the following cycle conditions: 94°C for 3 min, 45 cycles at 94°C for 15 s, 55°C for 15 s, 72°C for 30 s, and 72°C for 7 min. PCR products (15 μL) were analyzed by DGGE in an 8% polyacrylamide gel (1 mm thickness), using a parallel gradient of 30% urea-formamide (Wako Pure Chemical Industries) at the top of the gel and 70% at the bottom. Vertical electrophoresis was carried out using the DCode system (Bio-Rad Laboratories, San Diego, CA, USA) using 0.5× TAE buffer (20 mM Tris, 10 mM acetic acid, and 0.5 mM EDTA, pH 8.0) for 14 h at 100 V. The gel was stained in an ethidium bromide solution and then photographed on a UV transilluminator (ATTO, Tokyo, Japan).

### Histological examination of mouse distal colon samples

Sections of 6 μm thickness were prepared from Tissue-Tek-embedded colon samples using a Leica 2800E Frigocut Microtome Cryostat (Leica, Nussloch, Germany) and stained with hematoxylin and eosin (HE) [24]. Stained sections were examined under a microscope (E100; Nikon, Tokyo, Japan) and digitized images were taken at ×4 and ×40 magnification. Based on the histological score (HIS), consisting of the 5 indicators used by Liu et al. [24], the inflammation level of each section was evaluated.

### Real-time quantitative PCR (RT-qPCR)

Total RNA was extracted from colon tissue from each mouse using an RNeasy Mini Kit (QIAGEN, Hilden, Germany) according to the manufacturer’s instructions. cDNA was synthesized from 6.5 μg total RNA using a Reverse Transcription Kit (Takara Bio, Otsu, Japan). Quantification of mRNA expression levels was performed by applying SYBR Premix EX Tag II (Takara Bio) with a Thermal Cycler Dice Real Time System TP800 (Takara Bio) using specific primers for target genes (i.e., *TNF-α*, *COX-2*, *TLR4*, *Foxp3*, *IL-6*, *IL-1*, *SOCS3*, and *IL-17*) [24, 27]. The expression levels of the target genes were normalized to *GAPDH* as an internal standard [24]. The relative expression of the target genes was analyzed by the ΔΔCt method [10].

### High-performance liquid chromatography (HPLC)

Feces were suspend in sterilized water using a grinder pestle (SANSYO), then centrifuged (10 min at 20°C, 800 × *g*), and the supernatant was used as the analytical sample. After derivatizing the sample, the concentrations of acetic acid, pyruvic acid, and butyric acid in feces were measured by HPLC using a Hitachi LaChrom Elite HPLC System (Hitachi, Tokyo, Japan) and YMC-Pack FA 250 × 6 mm ID column (YMC, Kyoto, Japan) [28].

### Enzyme-linked immunosorbent assay (ELISA)

Colon samples were crushed using a grinder pestle (SANSYO), centrifuged (5 min at 20°C, 7500 × *g*), and the concentration of intestinal myeloperoxidase (MPO) in the supernatant was measured using an ELISA kit (Abcam, Cambridge, UK).

### Statistical analysis

Results are expressed as the mean ± standard deviation (S.D.). The statistical significance of the differences was determined using the Mann–Whitney U test. Differences were considered to be statistically significant when *p* < 0.05.

## Results and discussion

### Effects of DSS and each bacterium on disease activity

**Fig 2:**
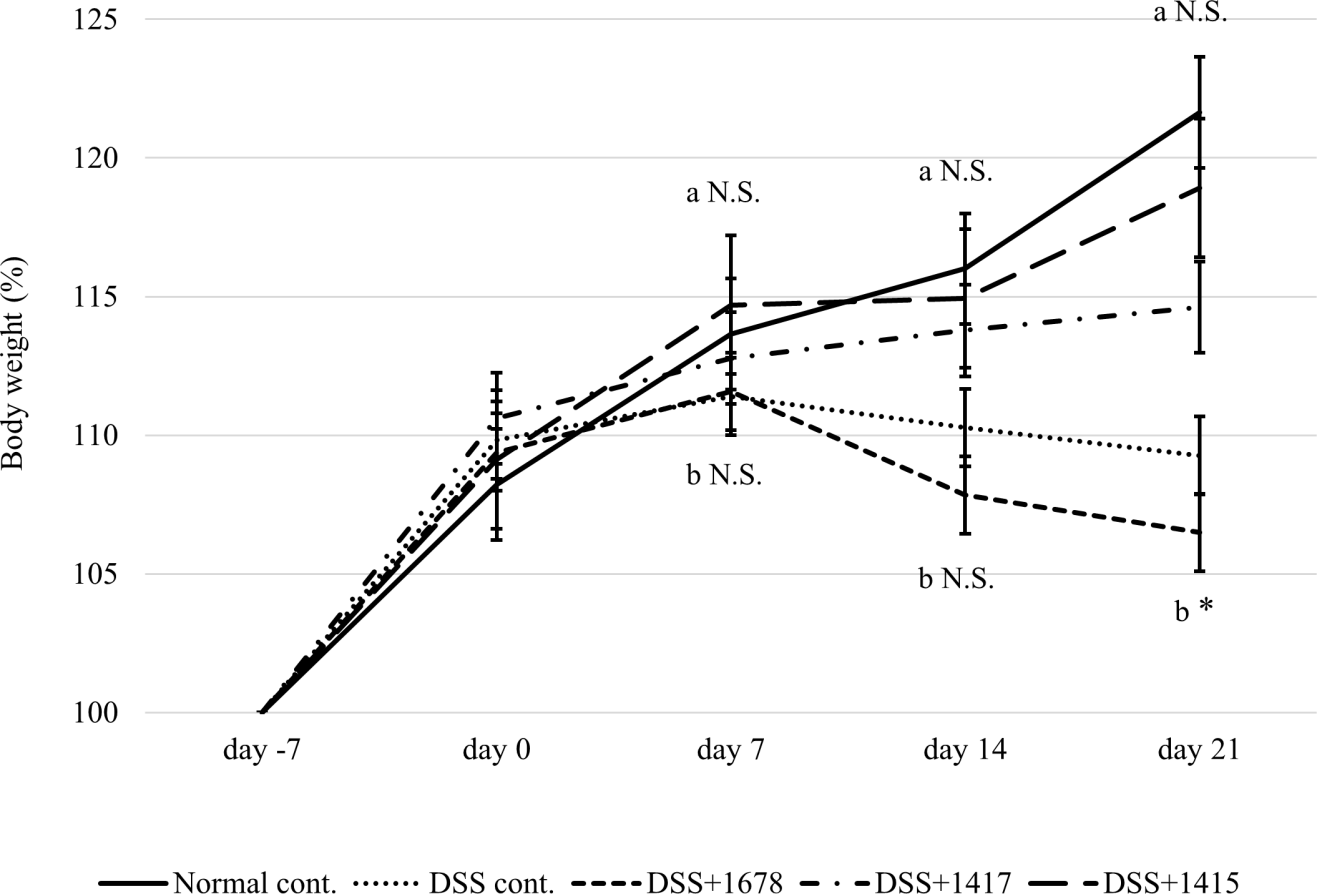
Rate of weight change in the mice. The mice were weighed every other week from day -7 to day 21 (*n* = 3 mice/group/period). The average weight of each group on day -7 was regarded as 100%. Data are expressed as the mean ± S.D. Differences between the comparison groups were considered statistically significant when *p* < 0.05 (*). N.S.: not significant; a: DSS+1415 group compared with Normal cont. group; b: DSS+1678 group compared with DSS cont. group.

To evaluate whether each bacterium affects mice, the administration of bacterial suspensions to mice was started from day -7. DSS treatment was started from day 0 to induce colitis. As shown in Fig 2, the degree of weight gain was similar in each group from day -7 to day 7. In the Normal cont., DSS+1415, and DSS+1417 groups, a weight gain rate of 10–20% was observed throughout the experiment. Conversely, in the DSS cont. and DSS+1678 groups, weight loss was observed after day 7, and the final weight gain rate remained at 5–10%.

**Fig 3:**
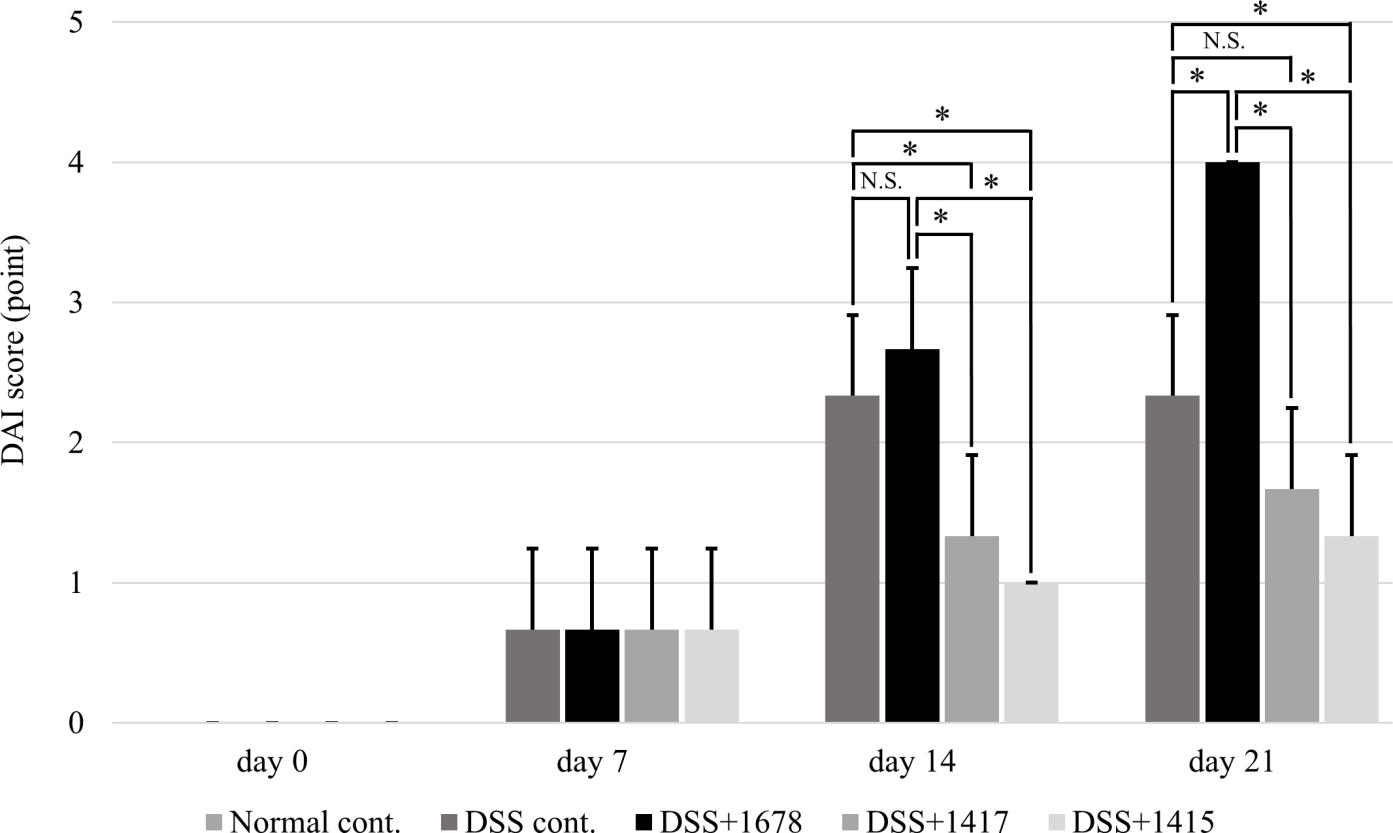
DAI in each group. The pathosis of each mouse was assessed based on the DAI score every other week from day 0 to day 21 (*n* = 3 mice/group/period). Data are expressed as the mean ± S.D. Differences between the comparison groups were considered statistically significant when *p* < 0.05 (*). N.S.: not significant.

The DAI score based on 5 indicators (Table 1) was used to evaluate the condition of each mouse. The score for each group was 0 points at day 0 (Fig 3), indicating that colitis could not be induced in the mice by the administration of each bacterial suspension for 1 week. Note that the DSS+1678 group (mean 4 points) had a higher DAI score than the DSS cont. group (mean 2.3 points) on day 21, suggesting that *P*. *bifermentans* PAGU1678 possesses the ability to exacerbate inflammation. Body weight loss could not be confirmed in all individuals of each group, and no individual showed a score of 3 in any index. The main contributor to the score of each group was stool softness or bloody stool, and it was confirmed that these measures were greatly influenced by DSS-induced colitis. The DSS+1415 group had an average score of 1/2–2/3 of that of the DSS cont. group from day 14 to day 21, in agreement with a previous report in which *L*. *plantarum* PAGU1415 was shown to reduce pathosis in a UC mouse model [24].

### Effects of DSS and each bacterium on fecal microbial diversity

**Fig 4:**
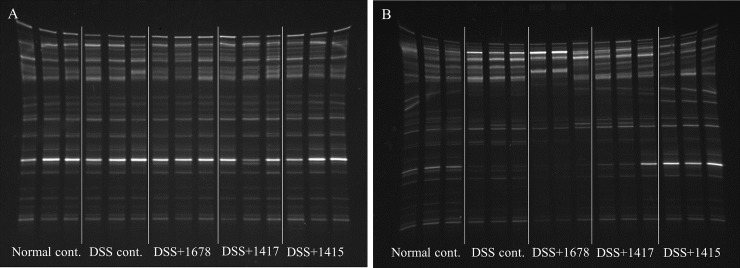
**Confirmation of intestinal bacterial diversity using DGGE analysis (A: day -7; B: day 21).** DGGE analysis was carried out using DNA extracted from the feces of 3 animals in each group. DGGE results on day -7 and day 21 showed a significant difference in intestinal constituent bacteria. The number of bands and each band density indicate the approximate number of bacterial species, and bacterial cells, respectively.

In order to investigate fecal bacterial diversity, DGGE analysis was performed using DNA extracted from feces (Fig 4). On day 0, there was no difference in the pattern of DGGE between the Normal cont. group and each DSS-treated group, but a reduction in the number of DGGE bands in the DSS-treated groups was observed on day 21. This result suggests that fecal microbial diversity was significantly reduced in the DSS-treated groups, consistent with a report that the diversity of intestinal flora in human UC patients is lower than that of healthy controls [11]. The number of DGGE bands in the DSS+1678 group was reduced as compared with that in the DSS treatment groups on day 21, suggesting that colitis was more severe in the DSS+1678 group than in the other groups. Meanwhile, the DSS+1415 group had the same bacterial composition as the Normal cont. group on day 21. The DAI scores also showed a similar tendency (Fig 3).

### Effects of DSS and each bacterium on histological assessment and colon length

**Fig 5:**
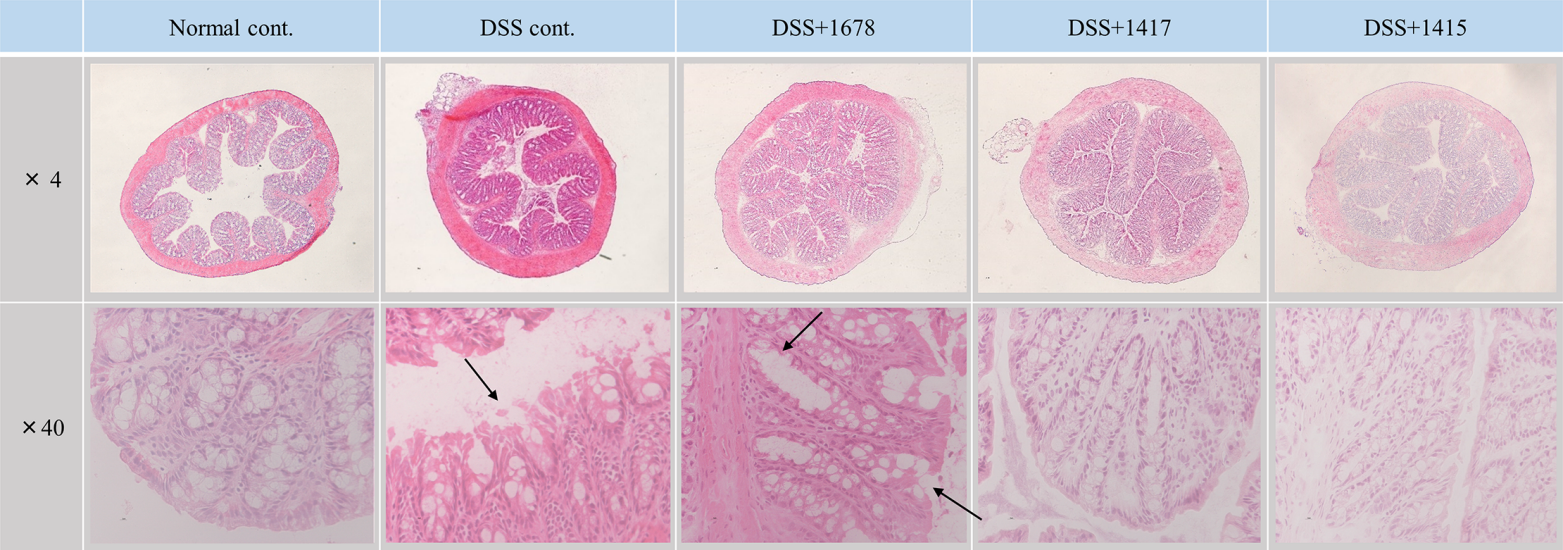
Representative HE-stained sections of mouse distal colon at day 21. All sections were digitized under ×4 and ×40 magnification and images were captured. Among the DSS treatment groups, especially in the DSS cont. group and the DSS+1678 group, mucosal layer damage and enterocyte loss were remarkable, and severe inflammation was observed (arrow).

**Fig 6:**
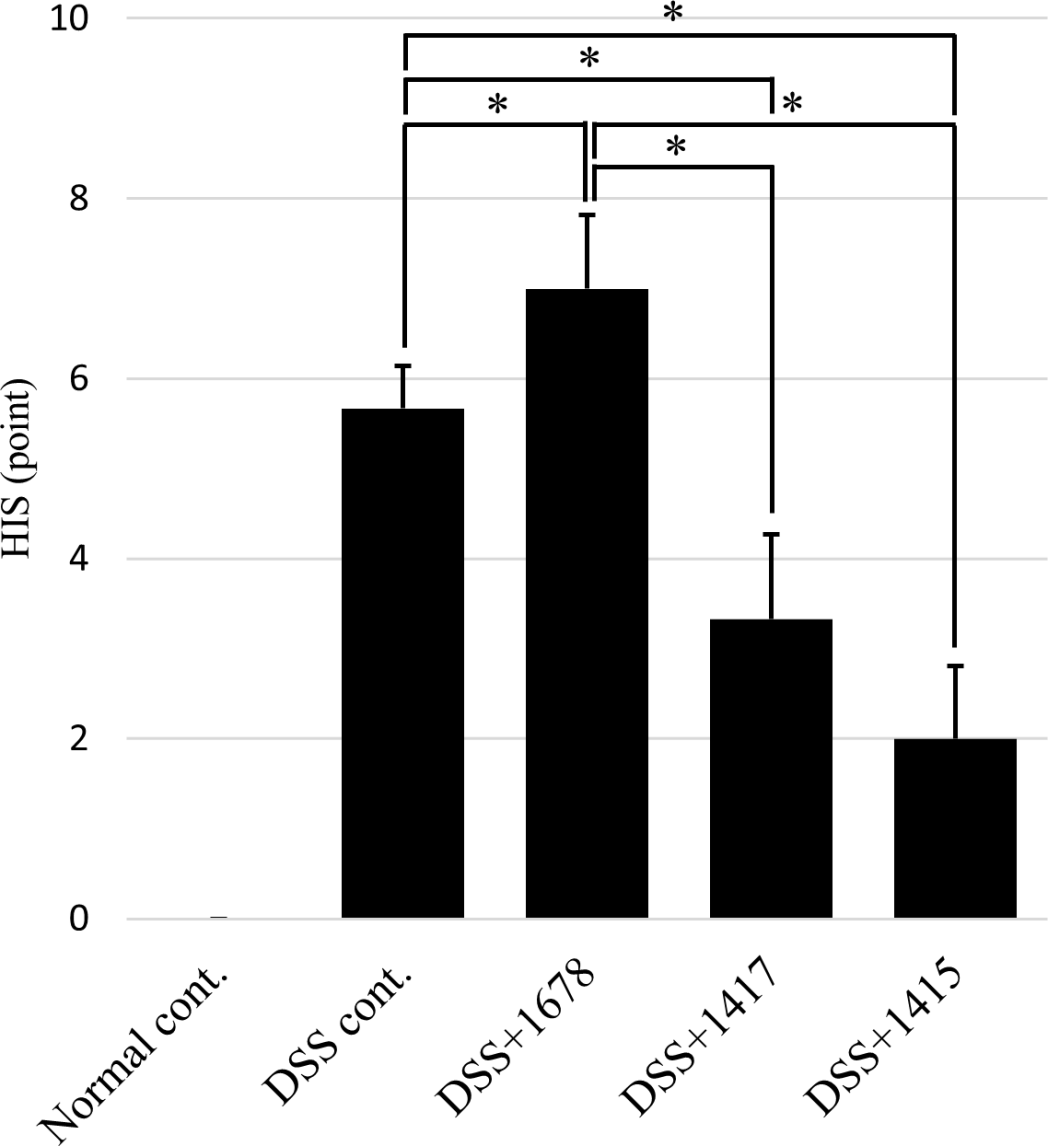
HIS on day 21. The HIS of each group was calculated from HE-stained images of each colon tissue sample (*n* = 3 mice/group). Data are expressed as mean ± S.D. Differences between the comparison groups were considered statistically significant when *p* < 0.05 (*). N.S.: Not significant.

In order to evaluate the influence of DSS or each bacterial suspension on the mouse intestinal tract, HE staining of colon tissue, calculation of HIS, and measurement of colon length were carried out. As shown in Fig 5, the disappearance of crypt structures and muscle layer thickening of the distal colon were observed in the DSS-treated groups on day 21. Especially, in the DSS cont. and DSS+1678 groups, since the mucosal layer was significantly irregular, exacerbation of colitis was confirmed visually as compared with the other groups. Furthermore, as a result of histological scoring, significant differences were detected between the DSS cont. and DSS+1678 groups (Fig 6). Meanwhile, in the DSS+1417 and DSS+1415 groups, there was no damage to mucosal structure, and their histological scores were approximately 1/2 of those in the DSS cont. and DSS+1678 groups. These results indicate that each bacterium may have a protective effect on colonic mucosa in DSS colitis.

**Fig 7:**
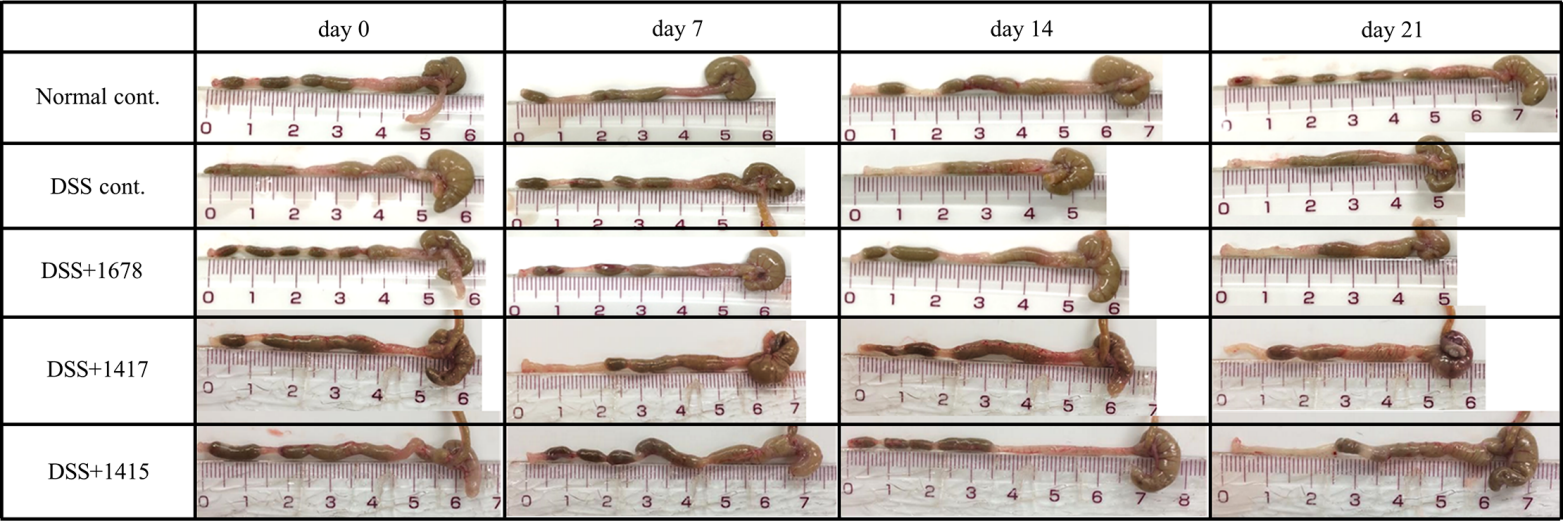
Morphological assessment of the colon of each mouse group. Colon tissue (cecum-rectum) obtained by autopsy every week from day 0 to day 21. A representative example of a colon with the average length from each group shown.

**Fig 8:**
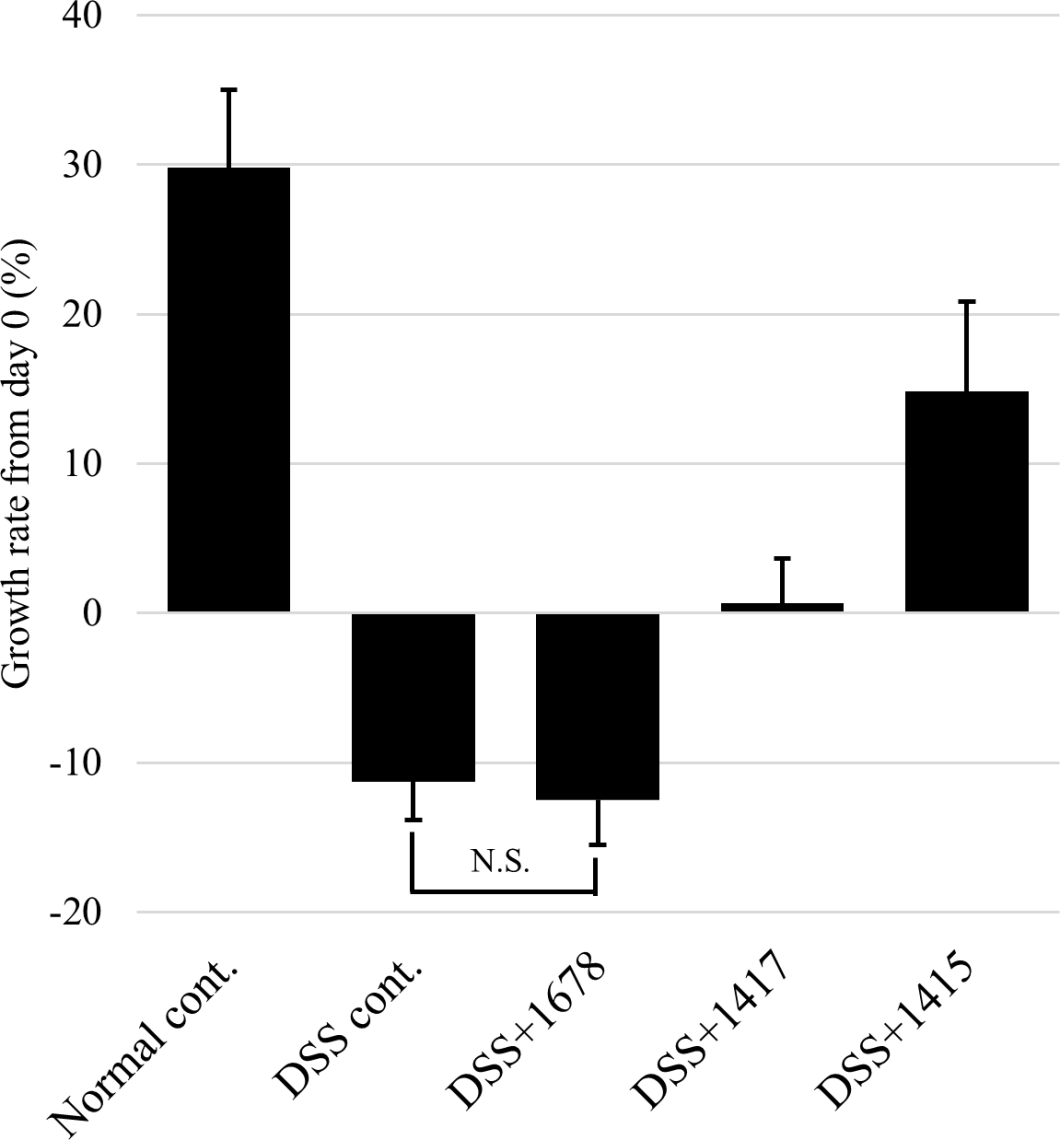
Growth rate of colon from day 0. The growth rate of the colon at day 21 was calculated based on colon length at day 0 (*n* = 3 mice/group). Data are expressed as mean ± S.D. Differences between the comparison groups were considered statistically significant when *p* < 0.05 (*). N.S.: Not significant.

At day 0, there was no difference in colon length between each group, but differences became visible depending on the treatment period of DSS or each bacterial suspension (Fig 7). In addition, the intestinal content of the DSS treatment groups became looser, and considering the results of the DAI score, confirmed the influence of DSS treatment on the exacerbation of pathosis. The growth rate in the DSS cont. and DSS+1678 groups on day 21, based on colon length at day 0, was remarkably decreased (Fig 8).

### Effects of DSS and each bacterium on the mRNA expression of factors related to inflammation and pro-inflammatory cytokines in the colon

**Fig 9:**
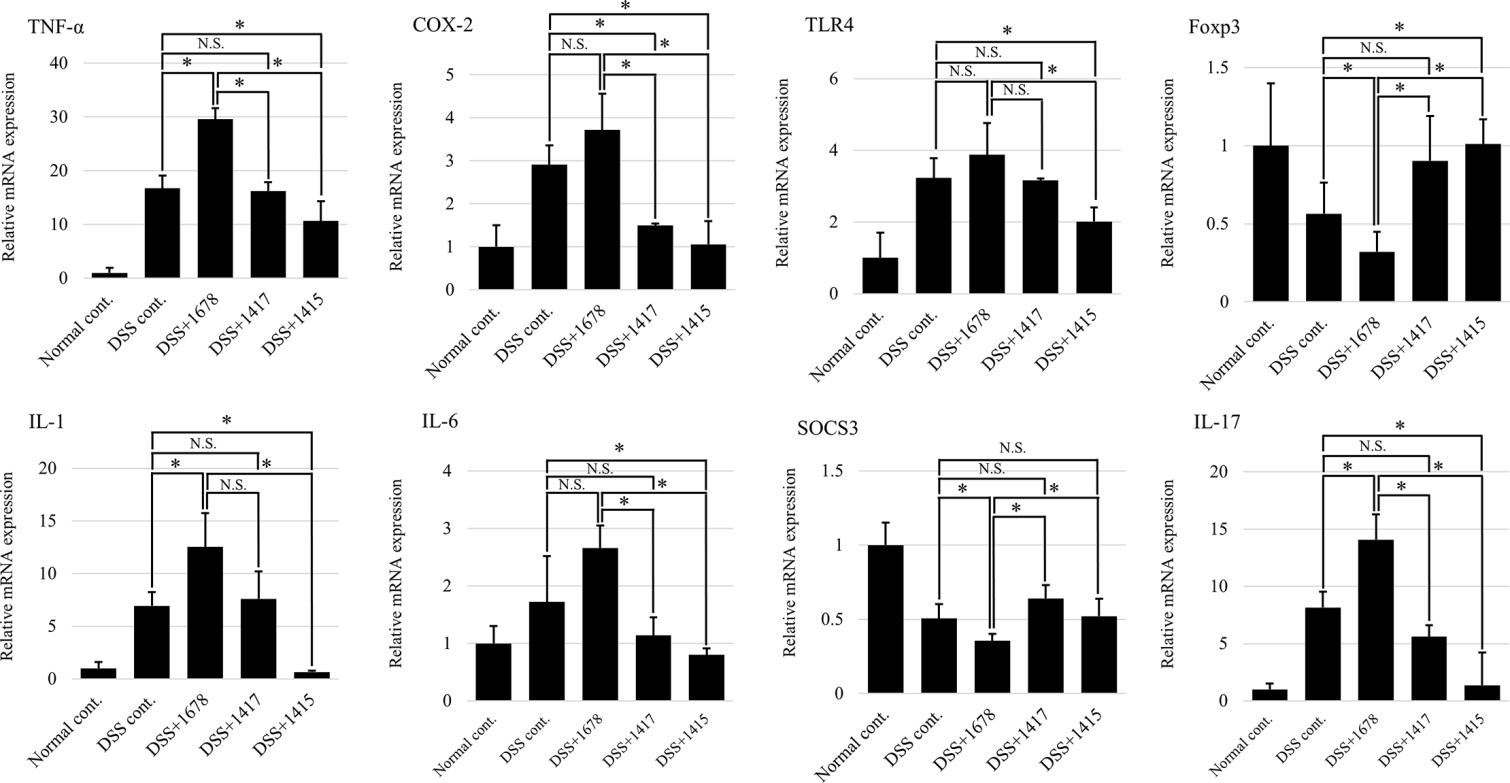
mRNA expression of factors related to inflammation and pro-inflammatory cytokines in the colon. RT-qPCR analysis was performed using day 21 samples, which showed the greatest differences among the groups, from the results of the assessment of mouse pathosis. The results are expressed as relative expression ratios to the Normal cont. group. Data are expressed as the mean ± S.D. (*n* = 3 mice/group). Differences between the comparison groups were considered statistically significant when *p* < 0.05 (*). N.S.: not significant.

To confirm the extent of intestinal inflammation at the cytokine level, the gene expression of inflammation-related factors (*COX-2*, *TLR4*, *Foxp3*, and *SOCS3*) and inflammatory cytokines (*TNF-α*, *IL-6*, *IL-1*, and *IL-17*) was analyzed by RT-qPCR. The results are expressed as the relative expression ratio relative to the Normal cont. group. As shown in Fig 9, the expression levels of *TNF-α*, *COX-2*, *IL-1*, and *IL-17* were increased by an average of 1.5-fold in the DSS+1678 group as compared with those in the DSS treatment groups. These results support a previous report in which the expression of *TNF-α*, *IL-1*, and so on was shown to be related to the severity of UC [29]. In contrast, in the DSS+1417 group, and especially the DSS+1415 group, their expression averaged 1.5–2-fold lower than in the DSS cont. group. As for *TLR4*, there was no statistically significant difference between the DSS cont., DSS+1678, and DSS+1417 groups. It is expected that all of the strains used in this study were Gram-positive bacteria and did not affect the activation of TLR4, which specifically recognizes lipopolysaccharide [30]. The expression of *Foxp3*, a master transcription factor that determines the differentiation and function of regulatory T (Treg) cells, was significantly reduced to less than 1/2 in the DSS+1678 group compared to the other DSS treatment groups. From this result, it is expected that Treg cells cannot control the excessive immunity (colitis) in the DSS+1678 group. The expression of *SOCS3*, which suppresses IL-17 production (specifically induced from Th17 cells during inflammation) [31], in the DSS treatment groups was reduced to an average of approximately 1/2 in comparison with the Normal cont. group, and the DSS+1678 group showed a maximum reduction of approximately 1/3. This result provides support for the observations that IL-17 expression is elevated in the lesion mucosa of IBD patients (including those with UC) [32] and that the severity of DSS-induced colitis is reduced in IL-17 knockout mice [33]. The results of these RT-qPCR analyses support the series of results showing exacerbation of colitis by *P*. *bifermentans* PAGU1678.

### Effects of DSS and each bacterium on the fecal concentrations of short chain fatty acids (SCFAs)

**Fig 10:**
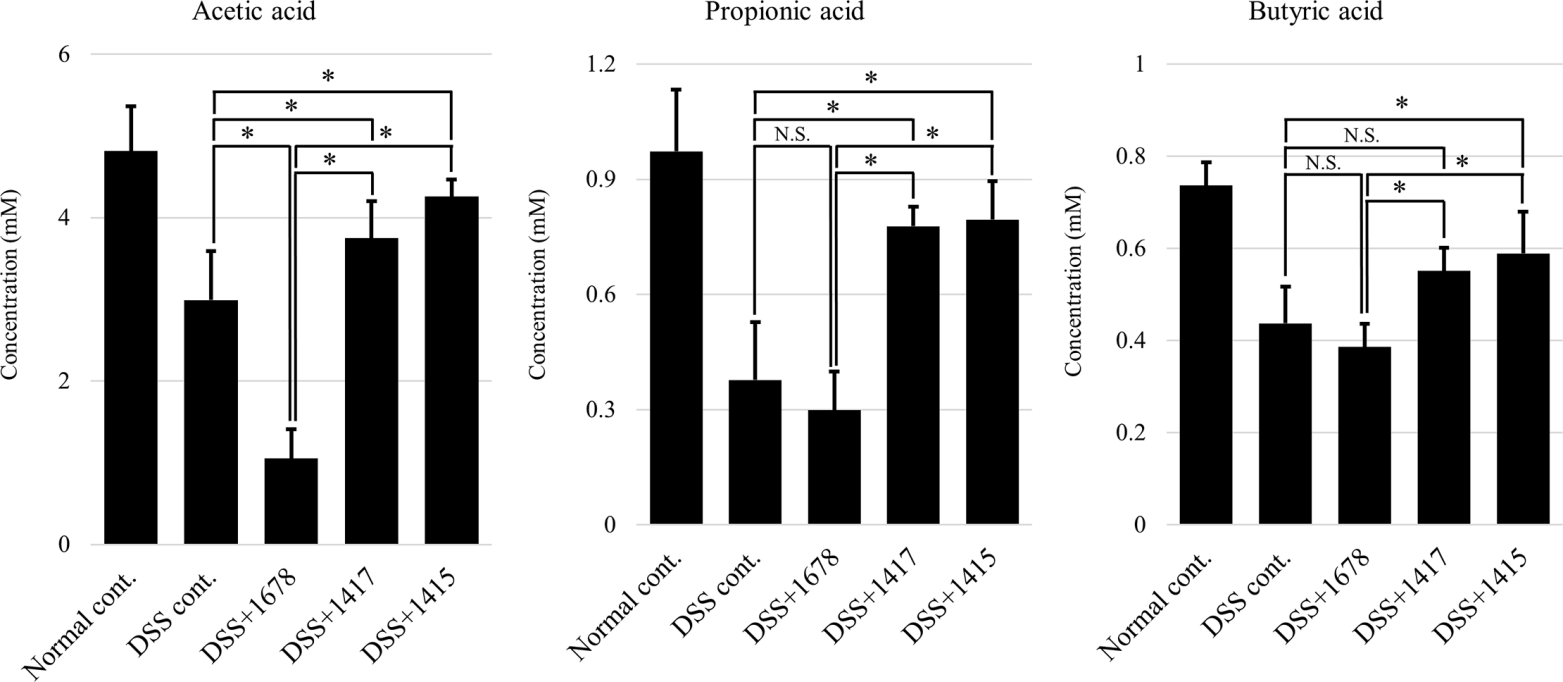
Concentrations of SCFAs in mouse feces. HPLC analysis was performed using day 21 samples, which showed the greatest difference among each group, from the results of the assessment of mouse pathosis. Data are expressed as the mean ± S.D. (*n* = 3 mice/group/period). Differences between the comparison groups were considered statistically significant when *p* < 0.05 (*). N.S.: not significant.

SCFAs (acetic acid, propionic acid, and butyric acid) produced by the fermentation of dietary fiber and oligosaccharides by intestinal bacteria are known to enhance the function of the intestinal barrier, induce Treg cells, alleviate mouse DSS-induced colitis, and remove mutated cells by the induction of apoptosis [34–39]. Therefore, the concentrations of each SCFA were measured in mouse feces at day 21. As demonstrated in Fig 10, in the DSS treatment groups, the production of each SCFA showed a remarkable reduction to a maximum of 1/2 of that in the Normal cont. group. Especially, in the DSS+1678 group, acetic acid production decreased to 1/2–1/3 of that in the other groups, propionic acid production was less than 1/2 of that in the DSS+1417 and DSS+1415 groups, and butyric acid production decreased to 2/3. These results show clearly that *P*. *bifermentans* PAGU1678 reduces the concentration of each SCFA by an unknown mechanism, such as an effect on intestinal symbiotic bacteria. The results for the production of butyric acid and the expression level of *Foxp3* (Fig 9) are consistent with a report that the abundant production of butyric acid by intestinal bacteria enhances *Foxp3* expression [36]. In other words, in the DSS+1415 group, in which DGGE analysis indicated that the intestinal flora was maintained (Fig 4B), butyric acid production increased by approximately 1.3-fold as compared with the DSS cont. group and the expression levels of *Foxp3* increased by approximately 2-fold.

### Effects of DSS and each bacterium on MPO activity in the colon

**Fig 11:**
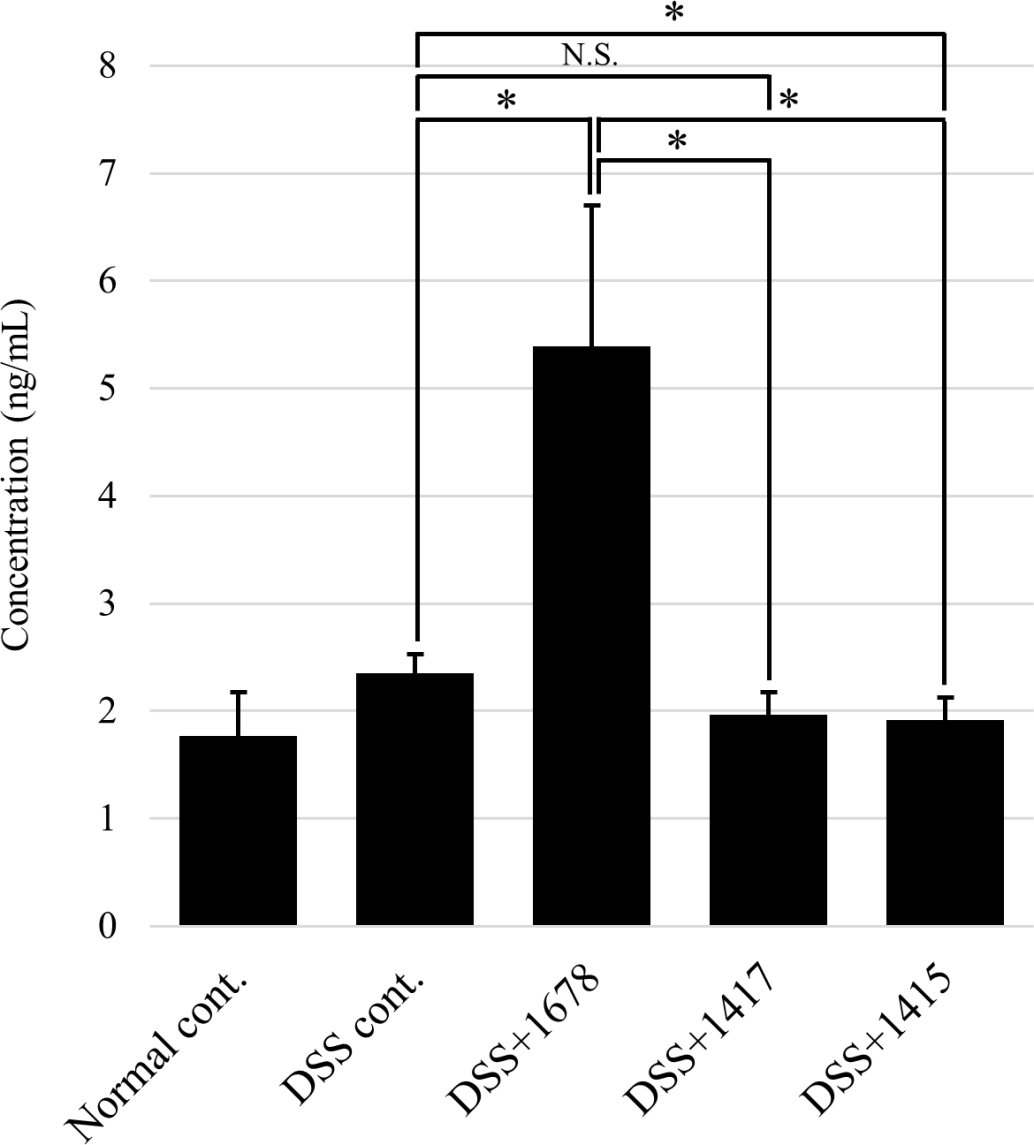
MPO activity in the colon. ELISA analysis was performed using day 21 samples, which showed the greatest difference among each group, from the results of the assessment of mouse pathosis. Data are expressed as the mean ± S.D. (*n* = 3 mice/group/period). Differences between comparison groups were considered statistically significant when *p* < 0.05 (*). N.S.: not significant.

The level of MPO activity is proportional to neutrophil concentration in inflamed tissues, and an increase in MPO activity is an indicator of neutrophil infiltration and inflammation [40, 41]. MPO activity was measured by ELISA (Fig 11). There was no significant difference between the Normal cont. group and the other DSS treatment groups, but MPO activity was significantly increased in the DSS+1678 group to more than twice the mean on average compared with the other groups. This result supports the series of results showing the exacerbation of colitis by *P*. *bifermentans* PAGU1678. Together with the results of the histological assessment of mouse colon by HE staining (Figs 5 and 6), neutrophil infiltration from epithelial cell injury sites in the DSS+1678 group is expected. TNF-α, IL-6, and IL-17 strongly promote neutrophil activity [42–44], and this was consistent with the observation that gene expression increased by more than 2-fold in the DSS+1678 group compared with the other groups (Fig 9).

## Conclusions

UC is an intractable intestinal disease for which the cause and fundamental therapy have not been established [45–48]. The gastrointestinal tract is inhabited by approximately 100 trillion bacteria of 500 species or more that maintain symbiotic relationships [49, 50]. When the intestinal bacterial flora is disturbed by diet, stress, and so on, and the number of detrimental bacteria increases, it is connected to a decrease in the number of probiotic bacteria such as *Lactobacillus* and *Bifidobacterium*, which have protective effects on the intestinal mucosa [51]. This study is the first to demonstrate experimentally the exacerbation of pathosis *in vivo* using a single bacterial strain in a mouse model of UC. In addition, we were able to discover a pathogenic bacterium from the class *Clostridia*, which is generally known as a group of bacteria contributing to the maintenance of health. We could not induce mouse colitis by administering only a *P*. *bifermentans* PAGU1678 bacterial suspension. This suggests that colitis is the result of the combined action of damage to the mouse intestinal mucosa layer by DSS and the induction of the inflammatory response by the invasion of this tissue by *P*. *bifermentans* PAGU1678. In other words, it is possible that *P*. *bifermentans* PAGU1678 is involved in colitis as a pathosis-exacerbating factor. By comparing the components involved in the immune response induced by *P*. *bifermentans* (formally known as *C*. *bifermentans*) and *C*. *butyricum*, pathosis-exacerbating factors could be determined.
